# Influence of Process Parameters in Graphene Oxide Obtention on the Properties of Mechanically Strong Alginate Nanocomposites

**DOI:** 10.3390/ma13051081

**Published:** 2020-02-28

**Authors:** Izaskun Larraza, Lorena Ugarte, Aintzane Fayanas, Nagore Gabilondo, Aitor Arbelaiz, Maria Angeles Corcuera, Arantxa Eceiza

**Affiliations:** 1 ‘Materials+Technologies’ Research Group (GMT) and Department of Chemical and Environmental Engineering, Faculty of Engineering, Gipuzkoa, University of the Basque Country (UPV/EHU), Pza Europa 1, 20018 Donostia-San Sebastian, Gipuzkoa, Spain; izaskun.larraza@ehu.eus (I.L.); aintzane.fa@gmail.com (A.F.); nagore.gabilondo@ehu.eus (N.G.); aitor.arbelaiz@ehu.eus (A.A.); 2 ‘Materials+Technologies’ Research Group and Department of Engineering Design and Project Management, Faculty of Engineering, Gipuzkoa-Eibar Section, University of the Basque Country (UPV/EHU), Otaola Hiribidea 29, 20600 Eibar, Gipuzkoa, Spain; lorena.ugarte@ehu.eus

**Keywords:** graphene oxide, size selection, sodium alginate, mechanical properties, thermal stability

## Abstract

Sodium alginate, a biopolymer extracted from brown algae, has shown great potential for many applications, mainly due to its remarkable biocompatibility and biodegradability. To broaden its fields of applications and improve material characteristics, the use of nanoreinforcements to prepare nanocomposites with enhanced properties, such as carbonaceous structures which could improve thermal and mechanical behavior and confer new functionalities, is being studied. In this work, graphene oxide was obtained from graphite by using modified Hummers’ method and exfoliation was assisted by sonication and centrifugation, and it was later used to prepare sodium alginate/graphene oxide nanocomposites. The effect that different variables, during preparation of graphene oxide, have on the final properties has been studied. Longer oxidation times showed higher degrees of oxidation and thus larger amount of oxygen-containing groups in the structure, whereas longer sonication times and higher centrifugation rates showed more exfoliated graphene sheets with lower sizes. The addition of graphene oxide to a biopolymeric matrix was also studied, considering the effect of processing and content of reinforcement on the material. Materials with reinforcement size-dependent properties were observed, showing nanocomposites with large flake sizes, better thermal stability, and more enhanced mechanical properties, reaching an improvement of 65.3% and 83.3% for tensile strength and Young’s modulus, respectively, for a composite containing 8 wt % of graphene oxide.

## 1. Introduction

In recent years, there is a growing interest in renewably sourced materials in the polymeric materials field, due to the fluctuations in oil price and the increase in environmental concern. Biopolymers, extracted from renewable sources, can be classified into three main groups: extracted from biomass, such as polysaccharides and proteins; synthesized from bioderived monomers, such as polylactic acid; and those produced by organisms or bacteria, such as bacterial cellulose [1,2]. Sodium alginate is an algae-based anionic and hydrophilic linear polysaccharide, characterized by its excellent biocompatibility, biodegradability, non-toxicity, and low cost [3,4]. Sodium alginate is a salt of alginic acid and is composed of (1→4)-β-D-mannuronic acid (M) and (1→4)-α-L-guluronic acid (G) units in the form of homopolymeric (MM or GG blocks) and heteropolymeric sequences (MG or GM blocks) depending on the source. It is extracted from brown algae (*Macrocystis pyrifera*) and can also be synthesized from microorganisms [5,6]. Alginate M and G units contain lateral hydroxyl and carboxyl groups that serve as reactive sites for several modifications [7]. Alginate is most commonly used in a water-soluble sodium alginate powder form, allowing physical cross-linking usually by the addition of divalent cations, such as Ca^2+^, Ba^2+^, and Zn^2+^ [7]. These divalent cations form a chelated structure, according to the commonly called egg-box model, preferentially with the GG blocks, thereby creating a stable three-dimensional network [8]. Alginate has potential in applications such as biomedicine, pharmaceutics, food industry, textiles, and additive manufacturing [9].

When compared to commercial synthetic polymers, the main drawbacks of alginate and biopolymers, in general, are their inferior thermal and mechanical properties [10], as well as their strong hydrophilic character. Currently, much effort has been made to improve the performance of alginates. In this sense, the addition of inorganic nanoreinforcements has been demonstrated to be an effective strategy to overcome the disadvantages of alginate [11,12,13], as well as to provide new functionalities. Gholizadeh et al. [14] incorporated different contents of hydroxyapatite nanoparticles to alginate and obtained an improvement in physical and mechanical properties, as well as antimicrobial activity, compared to alginate [14]. The addition of CuO nanoparticles to alginate improved the thermal properties and also conferred antifungal activity, which is valuable in biomedical applications [15]. The incorporation of silicon dioxide to alginate further reduced the water vapor permeability and swelling degree and significantly increased the mechanical properties, important parameters for packaging industries [16].

Similarly, when carbonaceous structures, such as graphene and graphene derivatives, are incorporated to alginate, not only do the mechanical and thermal behaviors improve and the hydrophilicity decreases, but nanocomposites with electrical conductivity and antibacterial properties are also achieved. Graphene is a 2D allotropic variety of carbon, constituted by a one-atom-thick layer of sp2-hybridized carbon atoms. It has been reported that a single-layered graphene exhibits a Young’s modulus of ∼1100 GPa, a tensile strength of 130 GPa, and electrical and thermal conductivities up to 6000 S/cm^−1^ and 5000 W m^−1^ K^−1^, respectively [17,18]. Graphene has the disadvantage of often being difficult to disperse in some biopolymeric matrices. Graphene derivatives, such as graphene oxide (GO), are more compatible due to the presence of hydroxyl, carboxylic, and epoxy groups on their surface and are consequently easier to disperse. GO is usually obtained by graphite oxide exfoliation through strong sonication treatments. Graphite oxide has been commonly synthesized by means of oxidative treatments and three main methods have been reported in the literature: Brodie’s method [19], Staudenmaier’s method [20], and Hummers’ method [21]. Among all of them, Hummers’ method and its modified method, are the most used since they are less toxic. Moreover, due to the hydrophilic character and to the presence of oxygenated groups at the surface of graphene oxide, it can be easily exfoliated and dispersed in aqueous media, being it compatible with most of aqueous soluble or dispersible polymers, this also has the benefit of being an environmentally. Liang et al. added 0.7 wt % GO to a poly(vinyl alcohol) matrix and observed an improvement of 76% and 62% in tensile strength and Young’s modulus, respectively [22]. Si et al. [23] biosynthesized a bacterial cellulose/graphene oxide nanocomposite (BC/GO) by adding GO to a culture medium. The BC/GO nanocomposite containing 0.48 wt % of GO showed an increase of 38% and 120% in tensile strength and Young’s modulus, respectively [23]. GO has also been incorporated into chitosan to prepare membranes for the removal of heavy metals and for evaluating their effect with respect to traditional chitosan and toxic glutaraldehyde membranes. An improvement in heavy metal adsorption has been observed with the addition of 5 wt % GO [24].

It has been observed that the properties of GO-containing nanocomposites are strongly dependent on the physical-chemical properties and morphology of GO, which are mainly influenced by the oxidation treatment and exfoliation degree. Long oxidation times lead to high contents of oxygenated groups and to high exfoliation degrees, which result in GO flakes with small size (several hundred nanometers) and thickness usually formed by a low number of layers (less than 2 nm) [25,26,27]. Thus, the interfacial interactions between GO and the matrix and hence, the final properties of the nanocomposite, will be influenced by the characteristics of GO and therefore, its preparation method.

There are many papers in the literature on the effect of graphene-obtaining treatment on the final properties of graphene, and quite a number of studies have assessed the effect of graphene content on the final properties of nanocomposites; however, not many studies are known, in which the effect of different types of graphene, with different characteristics depending on the treatment used to obtain them, on the final properties of nanocomposites is studied, and even less in the case of nanocomposites in which the matrix is a biopolymer. May et al. [28] concluded that the size of graphene flakes plays an important role in reinforcing polymers. They observed that when using large flakes and maintaining the thickness constant, the elastic modulus and tensile strength values of polyvinyl alcohol-graphene nanocomposites were significantly higher than that obtained by using smaller graphene flakes. Nawaz et al. [29] also observed that large flake sizes enhanced the elastic modulus and tensile strength, while elongation at break values diminished in polyacrylonitrile-graphene nanocomposites. Moreover, Szparaga et al. [30] observed a clear correlation between composite mechanical behavior and altered crystallinity in the structure, where mechanical properties of calcium alginate/GO composites improved with changes in crystallinity and average crystal area.

Therefore, this work focuses on obtaining graphene oxide with different characteristics for its subsequent incorporation (with varying content) to an alginate polymer matrix. In this sense, an extensive study of how oxidative treatment and graphene oxide isolation, assisted by centrifugation and sonication, affect its physical-chemical characteristics and morphology has been carried out. The effect of different sizes as well as thicknesses of graphene oxide flakes, determined by morphological studies, on the mechanical properties of nanocomposites has been analyzed.

## 2. Experimental Section

### 2.1. Materials and Methods 

Graphite flakes were purchased from Aldrich. Sulfuric acid (H_2_SO_4_, 96%), sodium nitrate (NaNO_3_, 99%), potassium permanganate (KMnO_4_, 99%), hydrogen peroxide (H_2_O_2_, 30% *w/v*) and hydrochloric acid (HCl, 37%) were supplied by Panreac (Barcelona, Spain). Medium viscosity alginic acid sodium salt from brown algae (SA powder, 4 Pa.s and M_ν_ = 2.4 × 10^5^ g mol^−1^, determined by viscosity measurements) was purchased from Sigma-Aldrich (St. Louis, MO, USA).

#### 2.1.1. Oxidation of Graphite

The oxidation process of graphite was carried out according to modified Hummers’ method [21]. Graphite flakes (1 g) were mixed with 0.5 g NaNO_3_ and 23 mL H_2_SO_4_ in an iced cooled bath at 0 °C for 30 min under continuous magnetic agitation. Then, 3 g KMnO_4_ was added to obtain a green-colored mixture. The mixture was kept at 0 °C for 2 h under magnetic agitation, until a purple color was achieved. Straightaway, it was heated at 35 °C in an oil bath for 30 min. Then, 46 mL of deionized water was slowly added. By this addition, the temperature of the mixture reached 98 °C. The mixture was kept at 98 °C for different times, 15 min and 30 min, and the samples thus obtained were designated as GO15 and GO30, respectively. The bath heater was shut down and 10 mL of H_2_O_2_ was added. The mixture was kept in the oil bath until the formation of bubbles stopped and it reached room temperature. Once at room temperature, 150 mL of deionized water was added. The resulting supernatant was discarded and a yellow-like mixture was obtained. The mixture was washed by centrifugation, using HCl (5 wt %) at 4500 rpm for 20 min. This step was repeated 5 times. Then, the same centrifugation procedure was repeated using deionized water until neutral pH was achieved. Finally, the mixture was filtered through polyamide filters (0.2 µm pore size, Sartorius, Göttingen, Germany) and was vacuum-dried at 50 °C for 48 h. Graphite oxide films, GO15 and GO30, were obtained.

#### 2.1.2. Exfoliation and Size Selection of Graphene Oxide

The obtained graphite oxide was exfoliated in water (0.5 mg mL^−1^), assisted by ultrasonication (Vibracell 75043, Sonics & Materials, Newton, MA, USA 30% amplitude). Ultrasonication times of 3 h and 4 h were applied for samples oxidized for 30 min, and these fractions were designated as GO30S and GO30L, respectively. The graphene oxide flakes, thus obtained, were size selected by centrifugation. Firstly, they were centrifuged at 4000 and/or 3000 rpm for 30 min and the supernatant fractions (ca. 80%) were collected. The fractions were named according to the applied ultrasonication and centrifugation treatments as GO30L-4000, GO30L-3000, and GO30S-4000. The remaining sediments of GO30L-4000 and GO30L-3000 fractions were collected and redispersed in water for 15 min using an ultrasonic bath. Centrifugation and redispersion steps were repeated for 2000 and 1000 rpm centrifugation rates to obtain size-selected graphene oxide flakes [31], denoted as GO30L-2000 and GO30L-1000, respectively.

The supernatant fractions were filtered through polyamide filters (Sartorius, 0.2 µm pore size) and dried for 48 h at room temperature. The designation of GO fractions and the applied oxidation and exfoliation procedures are summarized in Table 1.

#### 2.1.3. Preparation of Nanocomposites

First, dispersions of GO30L-4000 and GO30S-4000 fractions in water (5 mg mL^−1^) were prepared by ultrasonication for 1 h. Afterwards, sodium alginate/GO films were obtained. For this, sodium alginate in water at 2 wt % was used to incorporate different volumes of the GO dispersions previously prepared, so that the amount of GO was 1, 4, 6, and 8 wt % in the nanocomposites. The films were obtained by solvent casting and evaporating the water for 7 days at room temperature, and the samples were designated as SA-GO30L-4000-x% and SA-GO30S-4000-x%, where x is the percentage of GO in the nanocomposites. The samples were stored in a desiccator until their characterization.

### 2.2. Characterization Techniques

#### 2.2.1. Fourier Transform Infrared Spectroscopy

Fourier transform infrared (FTIR) spectroscopy was used to identify the characteristic functional groups of graphite, graphite oxide, and nanocomposites. Measurements were performed with a Nicolet Nexus FTIR spectrometer (Thermofisher Scientific, Waltham, MA, USA). For carbonaceous nanostructure characterization, KBr pellets (0.0025 mg sample g^−1^ KBr) were employed for the analysis. Single-beam spectra of the samples were obtained after averaging 32 scans in the range of 4000 to 400 cm^−1^, with a resolution of 2 cm^−1^. For composites, an MKII Golden Gate accessory (Specac) with a diamond crystal at a nominal incidence angle of 45° and ZnSe lens were used. Spectra were recorded in attenuated total reflection (ATR) mode between 4000 and 650 cm^−1^ with a resolution of 4 cm^−1^ and 32 scans.

#### 2.2.2. Ultraviolet-Visible Spectrophotometry

The absorbance of graphite and graphene oxide was measured by ultraviolet-visible spectrophotometry (UV-Vis), using open-top quartz cells. For sample preparation, low concentration dispersions were prepared (0.5 g sample mL^-1^ solvent) by using ethanol and deionized water for graphite and graphene oxide, respectively. The spectra were obtained in a UV-3600 UV-VIS-NIR spectrophotometer (Shidmazu, Kioto, Japan) in the wavelength range of 200 to 600 nm. 

#### 2.2.3. Raman Spectroscopy

Raman spectra of graphite and graphene oxide were obtained using a Reninshaw InVia (Renishaw, Wotton-under-Edge, UK) spectrometer, coupled to a Leica DMLM microscope (50x), with a laser of 514 nm wavelength (ModuLaser) operating at 5% of nominal potency. Data were collected in the range of 150 to 3200 cm^−1^. Values of exposure time and accumulations were set at 20 s and 5 respectively.

#### 2.2.4. X-Ray Diffraction

X-Ray diffraction (XRD) analyses were performed in a Philips X’Per PRO (Malvern Panalytical, Malvern, UK) automatic diffractometer, operating at 40 kV and 40 mA in theta-theta configuration. A secondary monochromator with radiation Cu-Kα (λ = 0, 154 nm) and the solid state detector PIXCEL (active length in 2θ: 3.347°) were used. Data were collected in the 2θ range of 5° to 70° (step size: 0.026 and time between steps: 60 s) in continuous mode.

The interplanar distance in the different samples was analyzed according to Bragg’s law [32,33]:

*nλ* = 2*dsinθ* (1)

where *n* is a natural number between 1 and ∞ (in this case, *n* = 1), λ is the wavelength of the X-rays used in the analysis (in this case, λ = 0.154 nm), *d* is the interplanar distance in crystal structure, and θ is the angle between the incident rays and the dispersion planes.

#### 2.2.5. Atomic Force Microscopy

The morphology of graphene oxide flakes was analyzed by atomic force microscopy (AFM). Height images were obtained in a Dimension Icon (Bruker, Billerica, MA, USA). scanning probe microscope equipped with a Nanoscope V controller (Bruker). Tapping mode was employed in air, using an integrated tip/cantilever (125 µm length with ca. 300 kHz resonant frequency). 

For sample preparation, GO fractions were dispersed in water (0.1 mg mL^−1^) using an ultrasonic tip for 1 h. A droplet of graphene oxide dispersion was put on a prewashed silicon wafer substrate and water was eliminated by spin coating at 1200 rpm for 120 s. Prior to analysis, samples were kept at room temperature for 48 h.

#### 2.2.6. Thermogravimetric Analysis

Thermal degradation of graphene oxide flakes and nanocomposites was assessed by thermogravimetric analysis (TGA) performed in a TGA/STDA 851 (Mettler Toledo, Columbus, OH, USA) equipment. Samples of around 5 mg were heated from room temperature to 700 °C at a heating rate of 10 °C min^−1^ under nitrogen atmosphere. 

#### 2.2.7. Mechanical Properties

Young modulus, tensile strength, and elongation at break of nanocomposites were analyzed in an Instron 5697 equipment (Instron, Norwood, MA, USA) using a load cell of 500 N in tensile mode. Tests were carried out at a crosshead speed of 2 mm min^−1^ and with an initial grip separation of 10 mm. Rectangular samples of 70 mm × 5 mm × 0.05 mm (length × width × thickness) were used. Tensile modulus (E), stress at yield (σ_y_), stress at break (σ_b_), and elongation at break (ε_b_) were determined from stress-strain curves and averaged from five specimens. 

## 3. Results and Discussion

### 3.1. Oxidation Process

**Figure 1:**
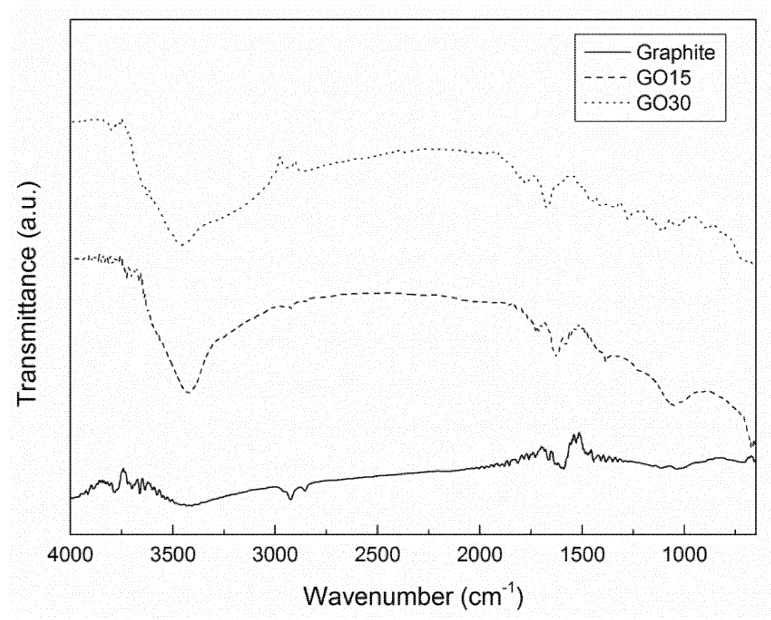
Fourier transform infrared (FTIR) spectra of graphite, GO15, and GO30 samples (y-axis of the curves were translated in order to avoid overlapping and to improve the visibility of the characteristic bands).

In order to study the different types of functional groups formed in the oxidation process of graphite as well as the effects of the different oxidation times, FTIR analysis was used. The FTIR spectra of graphite and GO are shown in Figure 1.

The peak corresponding to the stretching vibration of C=C bonds was clearly observed at around 1590 cm^−1^ [34] for graphite, while this peak was not so clear and could be overlapped in the case of oxidized graphite. Moreover, GO15 and GO30 samples showed additional peaks. The pronounced peak at around 3400 cm^−1^ was assigned to the stretching vibration of OH groups, derived from hydroxide and carboxylic acid groups, as well as from some moisture traces [35]. Moreover, a peak attributed to O–H bending can be seen at 1630 cm^−1^. The peak at 1735 cm^−1^ was assigned to the stretching vibration of C=O bonds in carboxylic acid [36]. Finally, a peak was also observed at 1050 cm^−1^ in graphite oxide samples, which was related to the stretching vibration of C–O–C [25]. These results confirmed the presence of oxygen-containing functional groups in graphite oxide, indicating that the oxidation process was carried out satisfactorily. No significant differences were observed for different oxidation grades of GO15 and GO30 samples. 

**Figure 2:**
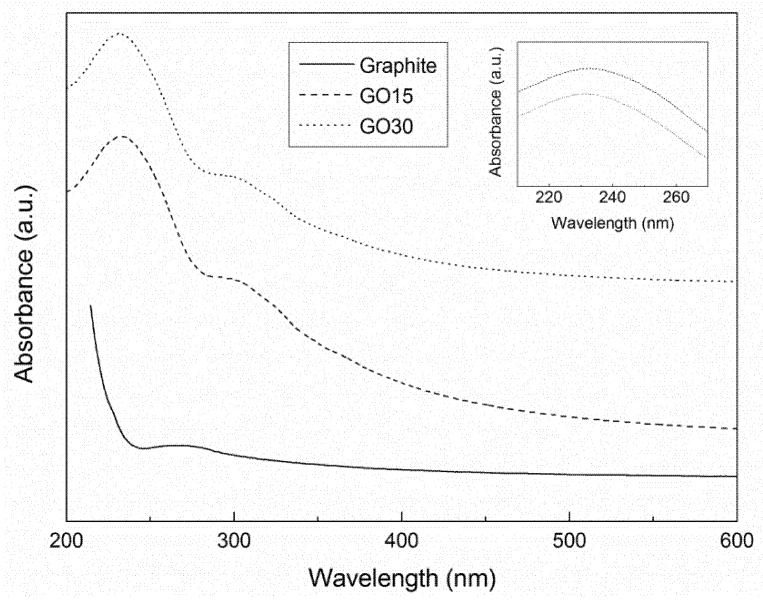
Ultraviolet-visible (UV-Vis) spectra of graphite, GO15, and GO30 samples.

The differences in oxidation grades of graphite oxide samples were analyzed by UV-Vis spectroscopy. The obtained spectra are shown in Figure 2.

The graphite sample did not show remarkable peaks. The spectra of graphite oxide samples showed two absorption maximums at around 230 nm and 300 nm. The peak around 230 nm was attributed to the π–π* transitions of aromatic C–C bonds, while the peak at 300 nm corresponded to the n–π* transitions of carbonyl (C=O) groups [25,37] and both can be bathochromically shifted by conjugation [38]. Both peaks were characteristic of graphite oxide, indicating that the oxidative process was effective, in good agreement with the FTIR analysis. 

The absorption peak of π–π* (C–C) transitions was studied in more detail (Figure 2, inset). It was observed that the peak appeared at 233 nm (wavenumber) in sample GO15, while it appeared at 231 nm in sample GO30. This shift in the wavenumber suggested that higher oxidation grades (GO30) resulted in a higher disruption of the structure of sp^2^ domain, thereby reducing the concentration of π electrons. As a consequence, more energy is needed for π–π* transitions [25,39].

**Figure 3:**
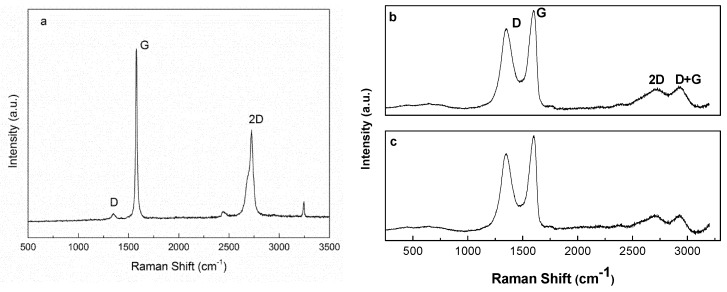
Raman spectra of (**a**) graphite, (**b**) GO15, and (**c**) GO30 samples.

Results of Raman spectroscopy analyses of graphite and graphite oxide are shown in Figure 3. All spectra showed typical G, D, and 2D bands associated with carbon materials [39]. The G band is assigned to the in-plane vibration mode due to the bond stretching of sp^2^ carbon pairs and the 2D band is related to the second order of zone-boundary phonons [39,40]. The D band is associated with flake edges since it needs a defect for activation [41,42]. In the case of graphite (Figure 3a), G, 2D, and D bands were observed at 1570, 2700, and 1354 cm^−1^, respectively.

In graphite oxide samples (Figure 3b), a shift to higher wavenumbers was observed for G peak compared to graphite. The maximum of the peak was observed at 1596 cm^−1^ and 1600 cm^−1^ wavenumber values for GO15 and GO30 samples, respectively. This shift to higher wavenumbers suggested a reduction of the in-plane sp^2^ domains as a result of the oxidation of graphite [43]. In the same fashion, an increase in the wavenumber of D band was observed in the GO30 sample (1348 cm^−1^ and 1351 cm^−1^ for GO15 and GO30, respectively). This may indicate the presence of more defects and disorders caused by hetero-atoms, grain boundaries, aliphatic chains, etc. as a consequence of stronger oxidation [35]. As shown in spectra b and c of Figure 3, beside the 2D band, a band located around 2920 cm^−1^, denoted as D + G band and related to defects, was also noted in the spectra of GO. For the 2D band, a slight decrease in intensity was observed in the GO30 sample, which can be explained by the breaking of the stacking order of graphene sheets along the z-axis due to oxidation [44]. These results confirmed stronger oxidation of the GO30 sample.

The relative intensity of D and G bands can be taken as indicative of crystallite size, according to the equation proposed by Cancado et al. [45]:

(2) $$L_{a}=\left[\left(2,4*10^{-10}\right){\left(\lambda_{1}\right)}^{4}\right]/\left[I_{\left(D\right)}/I_{\left(G\right)}\right]$$

where L_a_ is the average size of sp^2^ domain crystals, λ_1_ is the input laser energy, I_D_ is the intensity of D band, and I_G_ is the intensity of G band. 

The I_D_/I_G_ ratios and L_a_ values obtained for graphite, GO15, and GO30 samples are shown in Table 2. It was observed that, as the oxidation degree increased, the I_D_/I_G_ ratio increased, while L_a_ values decreased. This indicated that higher oxidation degrees resulted in smaller crystallites, the formation of defects, sp^3^ hybridizations, and changes in crystallinity [35,46]. In general, Raman results suggested that the structure of graphite was modified by oxidation. Variations due to different oxidation degrees were also observed, in good agreement with the UV-Vis analysis.

**Figure 4:**
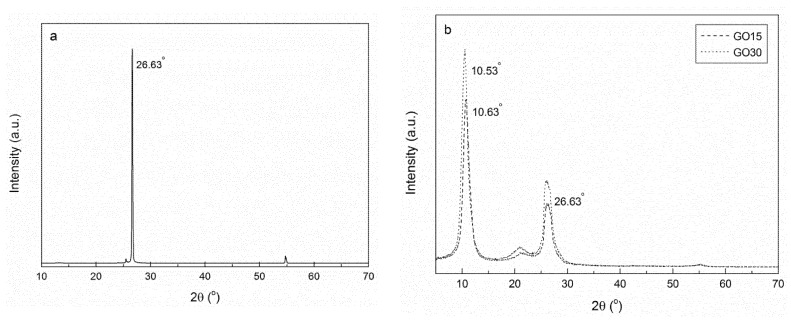
XRD patterns obtained for (**a**) graphite and (**b**) GO15 and GO30 oxidized samples.

The effect of different oxidation times on the interplanar distance of graphite was analyzed by XRD analysis. XRD patterns of graphite, GO15, and GO30 samples are shown in Figure 4. The XRD pattern of graphite structure showed a pronounced peak at 2θ = 26.63°, corresponding to the (002) plane of graphite [46] and taken as indicative of pure graphite [37]. This peak, although with lower intensity, also appeared in GO15 and GO30 samples, suggesting that total oxidation was not achieved. Furthermore, in oxidized samples, a new peak was observed at 2θ = 10.63° for GO15 and at 2θ = 10.53° for GO30, associated with a higher interlayer spacing, owing to the formation of more oxygen-containing functional groups on GO.

The distance between planes for the three systems was calculated using Equation (1) and the values obtained from the diffractograms (θ = 13.315°, θ = 5.315°, and θ = 5.265° for graphite, GO15, and GO30, respectively). Results are shown in Table 3. An increase in the interplanar distance was observed when comparing pure graphite with oxidized samples. These results suggested that the interplanar distance increased as a consequence of the insertion of functional groups containing oxygen and water molecules between the graphene oxide layers [34,37].

According to the results, it was concluded that the oxidation process was carried out satisfactorily and that the GO30 sample presented a higher degree of oxidation. Graphite residues were observed in the XRD patterns of GO15 and GO30 samples, indicating that a fraction of graphite was not oxidized. 

In view of these results, the sample GO30 was subjected to an exfoliation and posterior centrifugation process to eliminate the residual graphite fraction and obtain small-thickness graphene oxide flakes, according to the previously described procedure. The GO30 sample was selected due to the higher content of oxygen-containing hydrophilic groups that make graphite oxide easier to exfoliate in a polar medium.

### 3.2. Exfoliation and Size Selection of Graphene Oxide

To analyze the effect of sonication times (L: 4 h and S: 3 h) and centrifugation rates (4000, 2000, and 1000 rpm) on the characteristics of GO30 flakes, GO30L-4000, GO30L-2000, GO30L-1000, and GO30S-4000 samples were analyzed by XRD analysis and AFM. 

**Figure 5:**
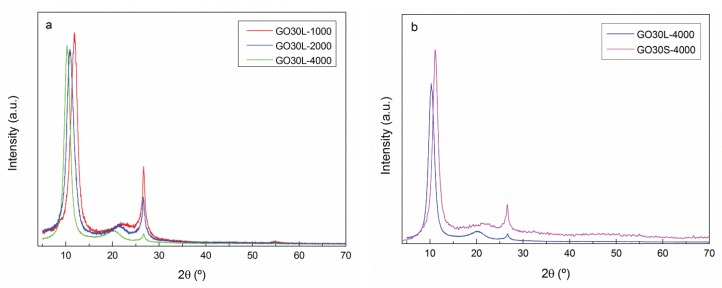
XRD analysis of (**a**) GO30L series and (**b**) GO30-4000 series.

XRD spectra of GO30 samples are shown in Figure 5. It was observed that, as centrifugation rate increased, the residual graphite content decreased. The intensity of the peak at 26.63° was the highest for the sample centrifuged at 1000 rpm and almost disappeared in sample GO30L-4000. This decrease in the intensity of the main peak corresponding to the plane (002) is related to a high level of exfoliation and disorder between GO flakes [37]. The distance between graphene oxide flakes, calculated according to Equation (2), increased with the centrifugation rate (Table 4). This indicated a higher level of exfoliation of the fraction GO30L-4000 [35]. For the effect of sonication times, it was observed that a shorter sonication time resulted in smaller distance between graphene oxide flakes, together with a higher fraction of graphite, in good agreement with a shorter exfoliation time.

**Figure 6:**
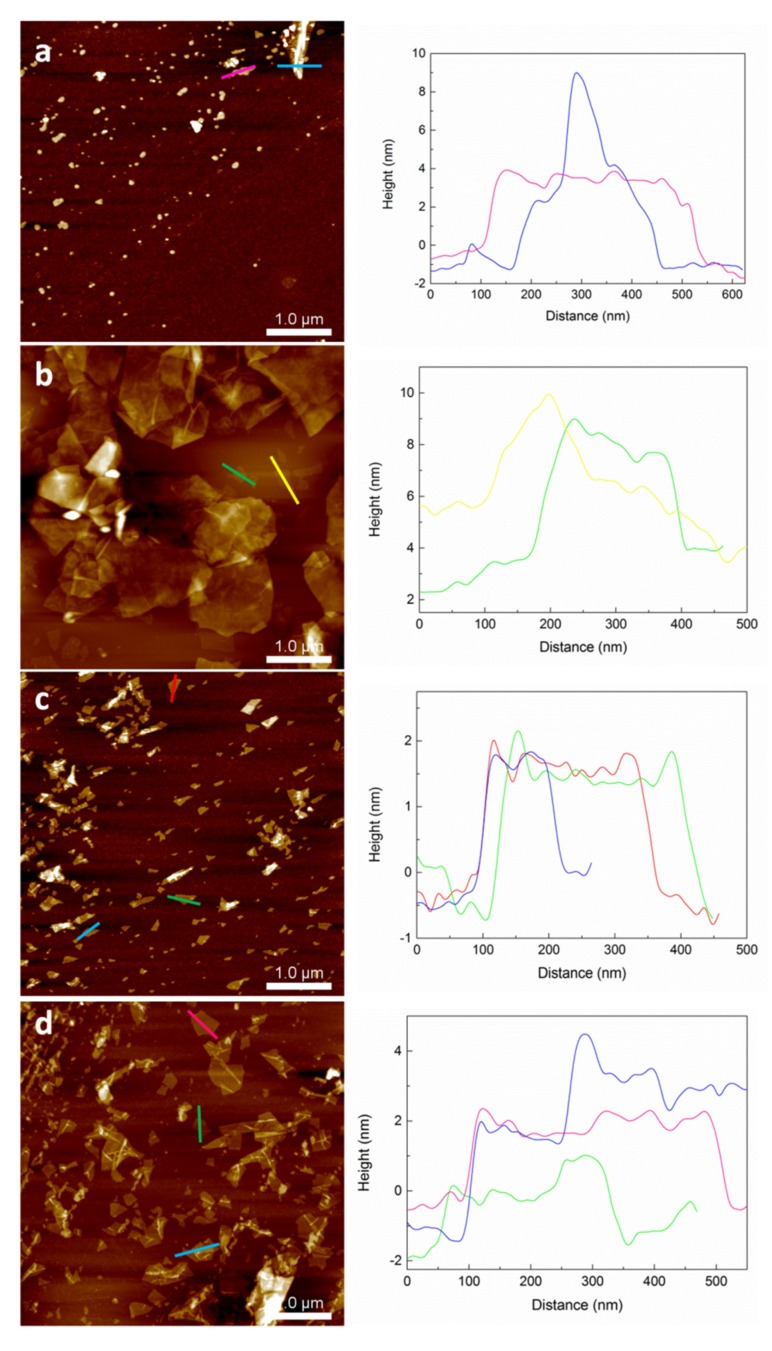
Atomic force microscopy (AFM) height images (left) and cross-sectional profiles (right) of (**a**) GO30L-1000, (**b**) GO30L-2000, (**c**) GO30L-4000, and (**d**) GO30S-4000 GO fractions.

The morphology of the GO flakes was observed by AFM and the results are shown in Figure 6. Concerning flake size, in GO30L-1000 and GO30L-2000 samples, irregular flakes of sizes between 300 and 500 nm were observed. In the GO30L-4000 sample, homogeneous flakes of size at around 250 nm were observed. A similar effect was observed in a previous work on centrifugation-based size selection of graphene [47]. Flake thickness was analyzed by cross-sectional profiles, and the GO30L-1000 and GO30L-2000 samples showed a heterogeneous distribution with values ranging from 4 to 10 nm. Flake thickness determined by AFM and the number of layers of graphene (*N*) can be related by Equation (3) [47,48]:

(3) $$N=\frac{t_{AFM}-0.4}{d_{spacing}}$$

where *t_AFM_* is the thickness measured by AFM, *0.4* is the factor that takes into account substrate–graphene and graphene–tip interactions, and d_spacing_ corresponds to interplane spacing in each sample. When this equation was applied to graphene oxide, measured thickness values for GO30L-1000 and GO30L-2000 fractions corresponded to multilayer GO flakes. For fraction GO30L-4000, thickness values at around 2 nm were observed, which may be related to few-layer graphene oxide flakes. 

Comparing the effect of ultrasonication time on flake morphology in GO30L-4000 and GO30S-4000 fractions (parts c and d of Figure 6, respectively), flakes of bigger size and similar thickness were observed when decreasing the ultrasonication time. Specifically, thickness values of around 3 nm and an average flake size of 450 nm were observed for GO30S-4000, whereas the thickness and size values were around 2 nm and 250 nm, respectively, for GO30L-4000. This suggested that ultrasonication time was related to the breaking of GO flakes, while thickness was more dependent on the centrifugation rate.

### 3.3. Nanocomposites

Considering the results obtained in graphene oxide characterization, sodium alginate-based nanocomposites were prepared with GO30L-4000 and GO30S-4000 graphene oxide samples. The most oxidized fraction (GO30) was chosen since the higher presence of oxygen-containing groups could favor the dispersibility of graphene oxide in water, as well as the interaction with the matrix. A centrifugation rate of 4000 rpm was chosen due to the presence of better exfoliated graphene flakes.

**Figure 7:**
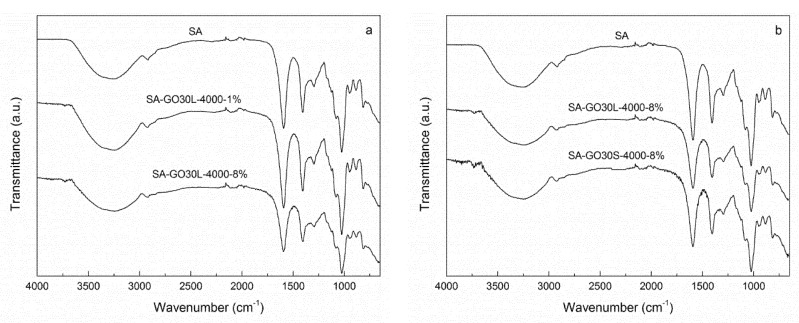
FTIR spectra of (**a**) SA (sodium alginate) matrix, SA-GO30L-4000-1%, and SA-GO30L-4000-8% nanocomposites and (**b**) SA matrix, SA-GO30L-4000-8%, and SA-GO30S-4000-8% nanocomposites (y-axis of the curves was translated in order to avoid overlapping and to improve the visibility of the characteristic bands).

FTIR spectra of the nanocomposites and pure SA matrix are shown in Figure 7. Figure 7a shows the spectra of SA-GO30L-4000-1% and SA-GO30L-4000-8%, as well as the SA matrix. No significant differences were observed when comparing the nanocomposites and the matrix. With the incorporation of graphene oxide, a slight broadening and a shift to lower wavenumbers were observed in the peak corresponding to the stretching vibration of the O–H bond. These changes would be an evidence of hydrogen-bonding interactions occurring between SA and GO. A similar behavior was observed when comparing SA-GO30L-4000-8% and SA-GO30S-4000-8% samples with the SA matrix (Figure 7b). No differences were observed with relation to different sonication times.

**Figure 8:**
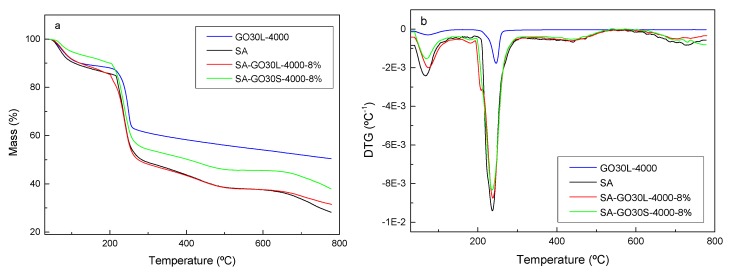
(**a**) Thermogravimetric (TG) and (**b**) derivative thermogravimetric (DTG) curves of GO30L-4000, SA matrix, GO30L-4000-8%, and GO30S-4000-8% nanocomposites.

Thermogravimetric (TG) and derivative thermogravimetric (DTG) results for the SA matrix and GO30L-4000, as well as SA-GO30L-4000-8% and SA-GO30S-4000-8% nanocomposites, are shown in parts a and b of Figure 8, respectively. The TG curve of GO showed that the thermal degradation process was carried out in three steps. In the first step, occurring between 25 °C and 100 °C, the mass loss was associated with the evaporation of water trapped between GO flakes [36]. The second step occurred between 230 °C and 260 °C and was related to the decomposition of the less stable oxygen-containing functional groups [36]. Finally, the slow mass loss observed from 260 °C was related to the more stable functional groups present in the graphene oxide, giving place to a high amount of residue [49]. The TG curves of nanocomposites showed a two-step degradation process. The first step, occurring at around 100 °C, was associated with the evaporation of water absorbed by the nanocomposite films. The second step, occurring between 200 °C and 300 °C, was related to the thermal decomposition of SA [26]. A higher amount of residue was observed for nanocomposites when compared with the matrix, which could be attributed to the excellent thermal stability of GO [50]. The sample SA-GO30S-4000-8% showed a higher amount of residue when compared with the SA-GO30L-4000-8% sample, suggesting that the bigger size of flakes obtained by shorter sonication times favored the thermal stability of final nanocomposites. 

Concerning DTG curves, shown in Figure 8b, it was observed that the temperature of maximum degradation rate slightly increased for nanocomposites with respect to the pure SA matrix, suggesting that the incorporation of GO enhanced the thermal stability and delayed the pyrolysis of nanocomposite films [51]. This increase in thermal stability in the presence of GO could be a result of interactions occurring between GO and SA. The presence of GO could hinder the mobility of SA molecular chains, increasing the energy required for thermal decomposition of nanocomposite films.

**Figure 9:**
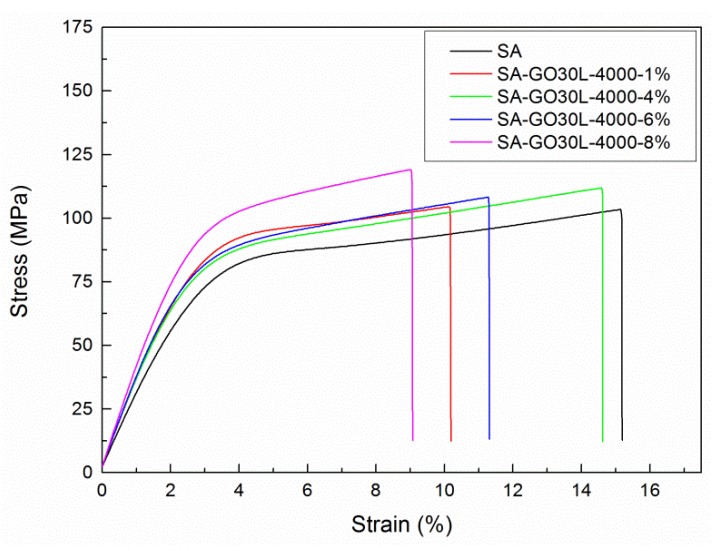
Stress-strain curves of SA-GO30L-4000 nanocomposite series with different GO30L-4000 content and pure SA matrix.

The effect of GO content in the mechanical properties of nanocomposites was assessed by tensile tests. Figure 9 shows the stress-strain curves of the SA matrix and SA-GO30L-4000 nanocomposite series, and their Young’s modulus, tensile strength, and elongation at break values are shown in Table 5.

Young’s modulus values of nanocomposites showed a slight increase with respect to the SA matrix. In general, an increase in tensile strength was observed with the incorporation of GO. Pure SA matrix showed a tensile strength of 101.4 MPa, while the maximum value obtained was 119.1 MPa for the nanocomposite with 8% of GO content. This resulted in an increase of 17.4% with respect to the matrix. The rest of the nanocomposites also showed improved tensile strength with respect to pure SA matrix. The improvement of mechanical properties was attributed to the uniform dispersion of GO in the SA matrix, as well as to the effective interfacial interactions, resulting in a good transference of stress from the SA matrix to rigid GO nanoreinforcements [26,50,51]. The elongation at break values obtained for the nanocomposites were lower than values obtained for the matrix. These results suggested that the addition of GO as a nanoreinforcement improved the strength and the stiffness of the films at the expense of flexibility.

**Figure 10:**
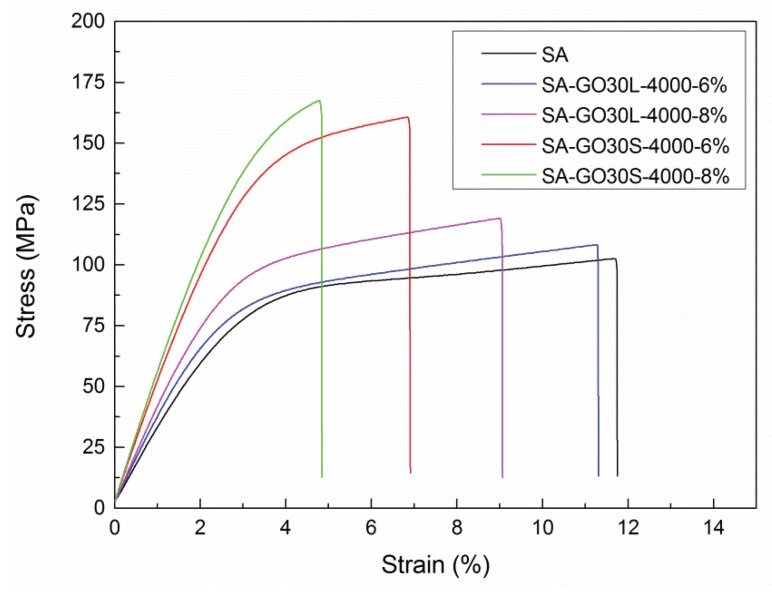
Stress-strain curves of SA-GO30L-4000 and SA-GO30S-4000 nanocomposites series as well as pure SA matrix.

To analyze the effect of GO flake size on mechanical properties, SA-GO30L-4000 and SA-GO30S-4000 nanocomposites series were compared, with a GO content of 6 wt % and 8 wt %. In AFM analysis, GO30L-4000 fraction showed thickness and flake size values around of 2 nm and 250 nm, respectively, while the thickness and flake size values for the GO30S-4000 fraction were around 3 nm and 450 nm, respectively. The stress-strain curves obtained for these samples are shown in Figure 10. Young’s modulus, tensile strength, and elongation at break values calculated from the stress-strain curves are listed in Table 6.

A significant increase was observed in Young’s modulus and tensile strength when the GO30S-4000 fraction was used as a nanoreinforcement. Concerning tensile strength, an improvement of 65.3% was observed for sample SA-GO30S-4000-8% when compared with the matrix (167.7 MPa vs. 101.4 MPa). For the SA-GO30S-4000-6% sample, an improvement of 58.8% was observed with respect to the matrix (160.8 MPa vs. 101.4 MPa), while for the SA-GO30L-4000-6% sample, an improvement of 6.7% was observed with respect to the SA matrix. Young’s modulus also increased significantly when the GO30S-4000 fraction was used. The values obtained for SA-GO30S-4000-6% and SA-GO30S-4000-8% nanocomposites were 5.1 GPa and 5.5 GPa, respectively, while for SA-GO30L-4000-6% and SA-GO30L-4000-8% nanocomposites, values of 3.6 GPa and 3.7 GPa were obtained, respectively. The elongation at break values diminished in the SA-GO30S-4000 series with respect to the SA-GO30L-4000 series. These results suggested that the effect of flake size is an important factor influencing the reinforcement effect when flakes of similar thickness are employed. Similar results were reported with other matrices by other authors [28,29].

**Figure 11:**
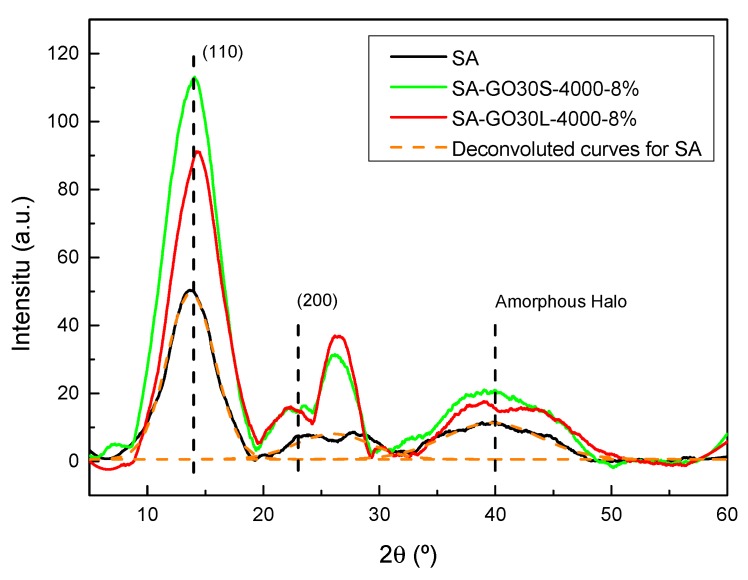
X-ray diffractograms for SA matrix, SA-GO30L-4000-8% and SA-GO30S-4000-8%, and deconvoluted curves for SA.

In order to assess the effect of the addition of GO and its size on the structure of alginate and, thus, in final composite properties, X-ray analyses were carried out. The resulting diffractograms are shown in Figure 11. The alginate diffractogram showed a very intense crystalline peak centered around 2θ = 13°, attributed to the (110), and a peak at 2θ = 22°, corresponding to the (200) plane. Regarding the amorphous zones, a broad peak can be seen in the diffractogram centered around 2 = 40° [52].

For composites, a clear increase in the intensity of the crystalline zones took place. A new peak centered around 2θ = 26° was also present, attributed to the (002) plane of the remaining graphitic structure of the carbonaceous reinforcements. In order to further analyze the system structure and crystallinity, numerical analyses were carried out to determine the crystalline phase content of each system. For that, diffractograms were deconvoluted with originPro9 using Gauss function and Equation (4) was used to calculate each value.

(4) $$X_{c}\left(\%\right)=\frac{A_{C}}{A_{C}+A_{A}}\;x\;100\;$$

where A_C_ is the sum of areas under the crystalline peaks and A_A_ is the area under the amorphous halo.

The crystallinity values for SA, SA-30GOL-4000-8%, and SA-30GOS-4000-8% were 74%, 78%, and 87%, respectively. The addition of GO resulted in more crystalline materials, which was greatly affected by the size of the flakes. Larger flakes, produced by shorter sonication times, resulted in systems with higher crystallinity degrees; this dependence on reinforcement shape has been previously reported by Szparaga et al. [30].

The crystallinity values were in agreement with mechanical behaviors shown by the samples. The higher crystallinity degree shown by reinforced composites, specially SA-30GO3-4000-8%, could explain the lower elongation at break values shown by these systems. This higher crystallinity could also add to the reinforcement supplied by the GO flakes in increasing the strength and Young’s modulus.

## 4. Conclusions

When graphite was subjected to the described oxidative process, it was observed that oxygen-containing groups were satisfactorily introduced into the graphitic structure. As the time of oxidative treatment increased (30 min vs. 15 min), a higher degree of oxidation, as well as a higher disruption of the graphitic structure, was observed. 

During the sonication and centrifugation processes to obtain graphene oxide, it was observed that higher centrifugation rates resulted in a graphene oxide fraction with lower amount of residual graphite. In the same fashion, graphene oxide flakes of lower size and thickness were isolated as centrifugation rate increased. According to AFM results, it was observed that, when using a centrifugation rate of 4000 rpm, few-layer graphene oxide flakes were obtained. When sonication time was decreased from 4 h to 3 h and final centrifugation rate was maintained at 4000 rpm, an increase in flake size was observed, while flake thickness values remained unchanged. In view of these results, it was concluded that ultrasonication time is related to the breaking of graphene oxide flakes, while flake thickness is more dependent on the centrifugation rate.

The nanocomposites showed evidences of hydrogen-bonding interactions between the SA matrix and graphene oxide, because of the oxygen-containing groups introduced during the oxidative process. The incorporation of GO increased the resistance to thermal degradation of the nanocomposites, probably due to the restrictions in SA chains motion as a consequence of interactions with graphene oxide. Higher flake sizes resulted in an improvement in the resistance to thermal degradation. In general, the incorporation of graphene oxide improved the tensile strength and Young’s modulus of the SA matrix. It was observed that, at similar graphene oxide thickness values, the increase in flake size significantly improved the mechanical properties.

## Figures and Tables

**Table 1:** Designation of graphene oxide (GO) fractions.

Sample	Oxidation Time (min)	Ultrasonication Time (h)	Centrifugation Rate (rpm)
GO15	15	-	-
GO30	30	-	-
GO30L	30	4	-
GO30S	30	3	-
GO30L-4000	30	4	4000
GO30L-3000	30	4	3000
GO30L-2000	30	4	2000
GO30L-1000	30	4	1000
GO30S-4000	30	3	4000

**Table 2:** I_D_/I_G_ ratios and L_a_ values for graphite, GO15, and GO30 samples.

Sample	I_D_/I_G_ Ratio	L_a_ (nm)
Graphite	0.063	264.4
GO15	0.81	20.7
GO30	0.85	19.8

**Table 3:** Interplanar distance (*d*) values calculated for graphite, GO15, and GO30.

Sample	2θ (°)	*d* (nm)
Graphite	26.63	0.335
GO15	10.63	0.828
GO30	10.53	0.837

**Table 4:** Interplanar distance (*d*) values for GO30L-1000, GO30L-2000, GO30L-4000, and GO30S-4000 samples.

Sample	2θ (°)	*d* (nm)
GO30L-1000	12.03	0.733
GO30L-2000	10.90	0.811
GO30L-4000	10.27	0.865
GO30S-4000	11.12	0.795

**Table 5:** Tensile strength, Young’s modulus, and elongation at break of SA-GO30L-4000 nanocomposite series and pure SA matrix.

Sample	Tensile Strength (MPa)	Young’s Modulus (GPa)	Elongation at Break (%)
SA	101.4 ± 4.8	3.0 ± 0.1	12.8 ± 3.9
SA-GO30L-4000-1%	104.5 ± 9.1	3.3 ± 0.3	10.2 ± 0.9
SA-GO30L-4000-4%	111.9 ± 15.1	3.2 ± 0.5	14.6 ± 2.9
SA-GO30L-4000-6%	108.3 ± 8.9	3.6 ± 0.3	11.3 ± 1.6
SA-GO30L-4000-8%	119.1 ± 6.2	3.7 ± 0.3	9.0 ± 3.0

**Table 6:** Tensile strength, Young’s modulus, and elongation at break of SA-GO30L-4000 and SA-GO30S-4000 nanocomposites series and pure SA matrix.

Sample	Tensile Strength (MPa)	Young’s Modulus (GPa)	Elongation at Break (%)
SA	101.4 ± 4.8	3.0 ± 0.1	12.8 ± 3.9
SA-GO30L-4000-6%	108.3 ± 8.9	3.6 ± 0.3	11.3 ± 1.6
SA-GO30S-4000-6%	160.8 ± 17.2	5.1 ± 0.7	6.9 ± 1.2
SA-GO30L-4000-8%	119.1 ± 6.2	3.7 ± 0.3	9.0 ± 3.0
SA-GO30S-4000-8%	167.7 ± 17.4	5.5 ± 0.3	4.8 ± 0.8
